# Myocardial Infarction in a Premenopausal Woman on Leuprolide Therapy

**DOI:** 10.1155/2015/390642

**Published:** 2015-06-23

**Authors:** Irving E. Perez, Mark A. Menegus, Cynthia C. Taub

**Affiliations:** Division of Cardiology, Montefiore Medical Center, Albert Einstein College of Medicine, 1825 Eastchester Road, Bronx, NY 10461, USA

## Abstract

Premenopausal women with chest pain syndrome may have nonatherosclerotic coronary arteries with abnormal coronary flow. Estrogens have cardioprotective effect improving coronary vasodilatation. This case report discusses the consequences of leuprolide use by decreasing estrogen levels which led to acute myocardial infarction.

## 1. Introduction

The leading cause of death among American women is coronary vascular disease (CVD). Approximately one of every 6 deaths in the United States is attributable to CVD [1]. Traditionally women are thought to be at lower CVD risk than men, with an onset of CVD approximately 10 years later [2]. The incidence of ischemic heart disease is lower in women than in men until menopause, after which their cardiovascular risk rises to equal that of men. This suggests that estrogens may have a cardioprotective effect [3, 4]. Leuprolide, an agonist of gonadotropin releasing hormone (GnRH), acts as a potent inhibitor gonadotropin secretor, decreasing ovarian and testicular steroidogenesis with subsequent decrease in estrogen and testosterone levels. It is used for uterine leiomyomata (fibroids), endometriosis, prostate cancer, and central precocious puberty. In premenopausal women on leuprolide therapy, estrogen is reduced to postmenopausal levels. Leuprolide is known to cause ischemic heart disease and angina in men [5]. To our knowledge, only two cases of cardiovascular disease secondary to leuprolide use have been reported in women [6, 7]. In this report, we describe a premenopausal woman on leuprolide therapy who presented with ST segment elevation (STE) myocardial infarction.

## 2. Case Report 

A 43-year-old premenopausal woman with a past medical history of hypertension and uterine fibroids accompanied by heavy uterine bleeding and subsequent iron deficiency anemia presented to the emergency department complaining of chest pain (CP). Electrocardiogram (ECG) and troponins were within normal limits and the patient was discharged home. Her only medications were hydrochlorothiazide, leuprolide, and ferrous sulfate. The following day the pain recurred, waking her from sleep. It was retrosternal, pressure-like, and radiated to left arm associated with diaphoresis, nausea, and vomiting, which continued to worsen. The pain became unbearable; emergency medical services were activated.

She denied any personal or family history of cardiac disease but has 2 cousins with venous thromboembolism (one with provoked deep venous thrombosis (DVT) after surgery and the other with recurrent unprovoked DVT/PE on lifelong anticoagulation). There were no recent upper respiratory infection symptoms, sick contacts, or travel history. She had 1 miscarriage at 3 months attributed to abnormal uterine anatomy, which was repaired surgically; she had two subsequent uneventful pregnancies, both delivered by C-section. She does not smoke cigarettes and her review of symptoms since starting leuprolide is positive for weight gain, sweats, and hot flashes.

**Figure 1:**
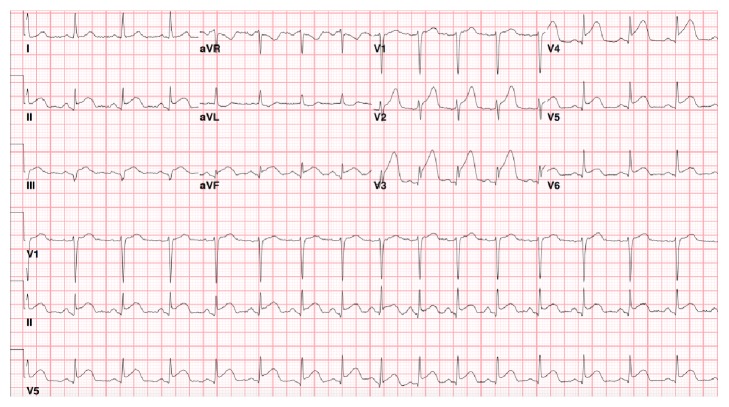
Initial ECG showing ST elevations in V2–V5, II, III, and AVF which are consistent with anterolateral and inferior STEMI.

On physical examination, the patient was anxious and in distress secondary to chest pain. She was hemodynamically stable. The ECG revealed anterolateral and inferior ST segment elevations (Figure 1). She received Aspirin 325 mg, Clopidogrel 600 mg, and Heparin 5000 U in the ED and was sent for emergent cardiac catheterization.

**Figure 2:**
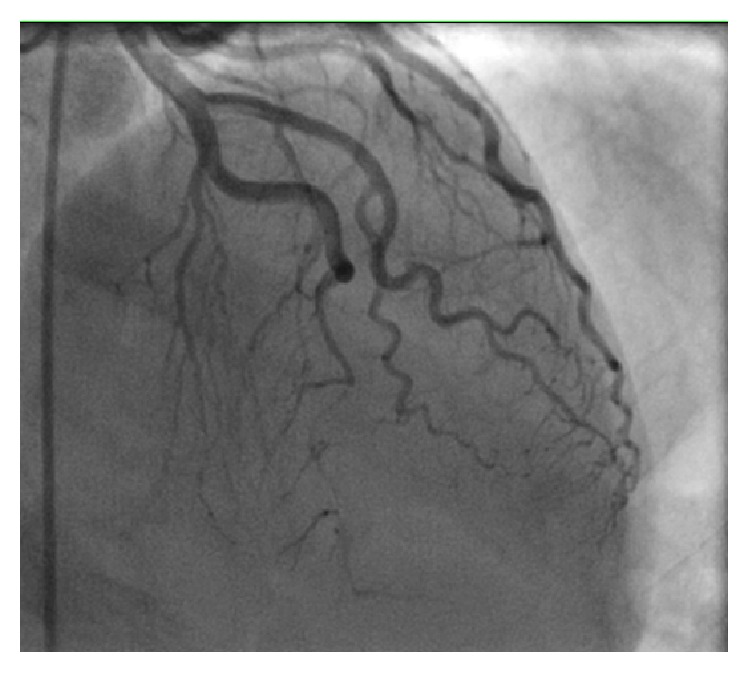
Cardiac catheterization demonstrating severe mid to distal LAD occlusion.

**Figure 3:**
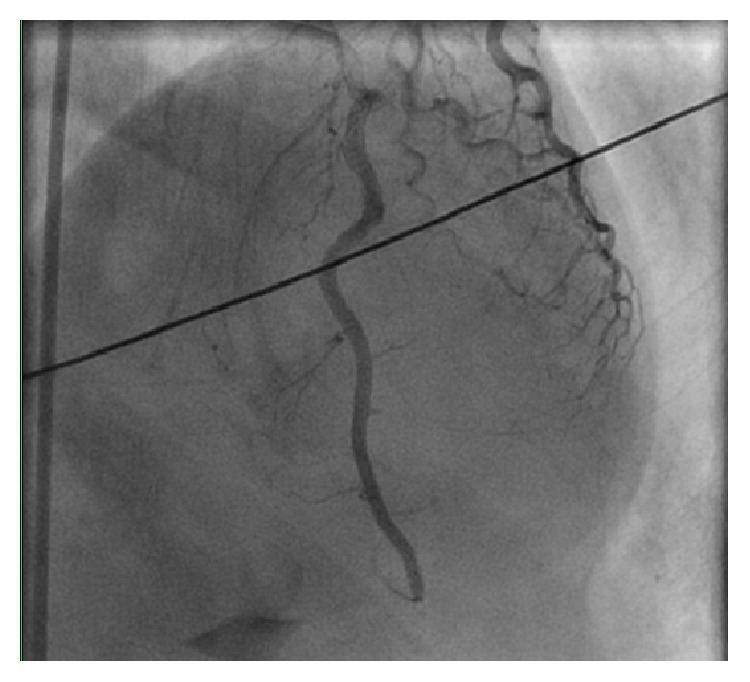
Cardiac catheterization after stent placement showing return of the blood flow in the LAD territory.

She was found to have a long, tortuous 99% mid and distal left anterior descending (LAD) lesion with Grade I flow (Figure 2). The entire segment was dilated with 1.5 and 2.0 mm balloons and stented with three Zotarolimus-eluting stents, two 2.25 × 30 mm and one 2.25 × 18 mm, and postdilated with 2.0 mm and 2.75 mm balloons with Thrombolysis in Myocardial Infarction (TIMI) III Grade flow restored (Figure 3). The right and left circumflex coronary vessels were angiographically normal.

During cardiac cath. the patient had a brief episode of ventricular fibrillation, less than 5 seconds, that self-resolved. The door-to-balloon time was 75 minutes. Troponin was 0.04 ng/mL on admission, which peaked at 2.68 ng/mL.

Aspirin and Clopidogrel were continued and Atorvastatin and Metoprolol were added. Echocardiogram showed anteroapical akinesis with a left ventricular ejection fraction of 40–45%.

**Figure 4:**
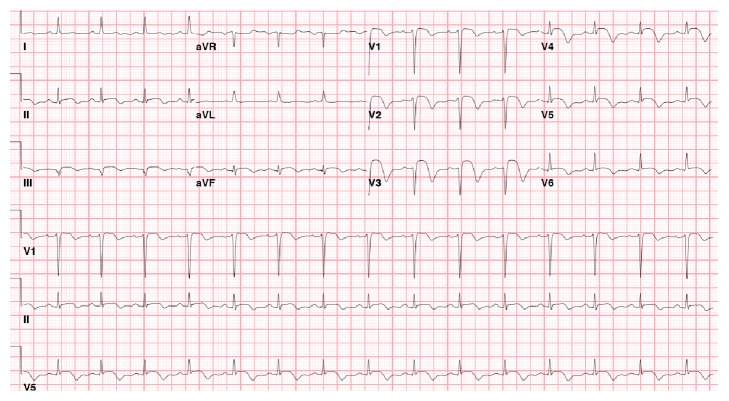
EKG after showing resolution of STE now shows new STE in V3-V4 while the patient was complaining of recurrent CP approximately 15 hours after cardiac catheterization.

**Figure 5:**
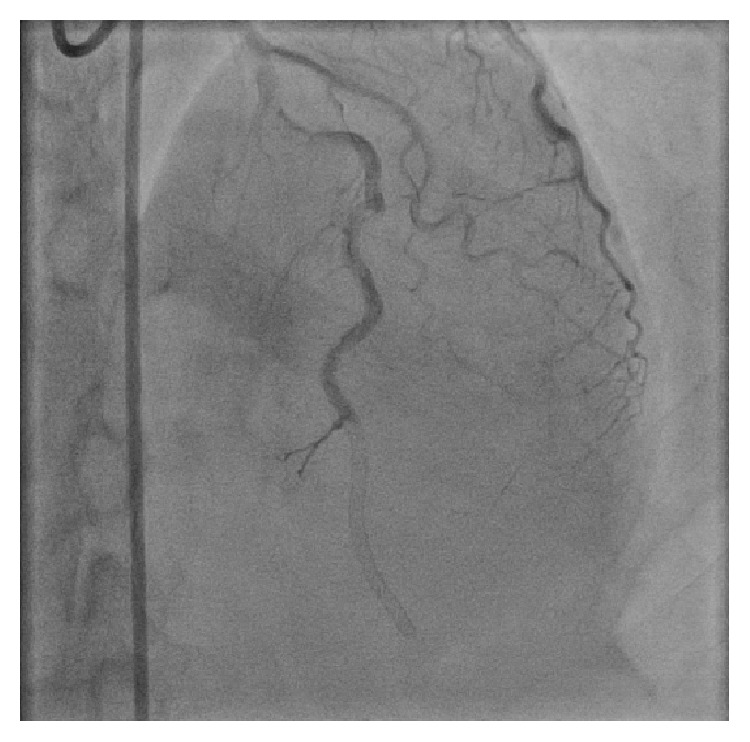
After second intervention cardiac catheterization showed distal LAD stent thrombosis with TIMI Grade 0 flow.

Fifteen hours after cardiac catheterization the patient developed recurrent CP partially alleviated with nitroglycerin and Morphine. There were new 2-3 mm STE in ECG leads V2–V5 (Figure 4). She was started on Heparin and Abciximab. The presumptive diagnosis was acute stent thrombosis (ST) and she was taken to the cath. lab immediately with a plan to acutely reopen the LAD and examine it with intravascular ultrasound (IVUS). The mid LAD stent was patent, but there was 100% thrombosis of the distal LAD stents (Figure 5). Attempts to pass various guidewires and a 1.5 mm undilated balloon through the stents were unsuccessful. The procedure finally was abandoned due to concern about potential wire fracture. Heparin drip was restarted, Metoprolol was increased, and Isosorbide Mononitrate and Lisinopril were added. Hematology was consulted due to concern for thrombophilias.

Serology for Paroxysmal nocturnal hemoglobinuria, Janus kinase 2 mutation, and Antiphospholipid antibodies were negative. She had no prior Heparin exposure making this presentation inconsistent with Heparin-induced thrombocytopenia. The only serologic abnormalities were antinuclear antibody 1 : 32 (fine speckled pattern) and anti-SSA/RO > 8.0 EU/mL.

It was believed that leuprolide may have been the proximate cause, this medication was discontinued, and the patient underwent uterine artery embolization without complications. The patient was discharged and followed up twice within 6 months without complications; labs only showed iron deficiency anemia.

## 3. Discussion 

The patient presented with STE myocardial infarction while on leuprolide therapy. The culprit lesion was a long, mid, and distal LAD near-occlusion. Women with chest pain syndromes are more likely than men to have nonatherosclerotic coronary arteries with abnormal coronary flow. There are various forms of nonatherosclerotic coronary artery disease (NACAD), including coronary vasospasm, dissection, fibromuscular dysplasia, ectasia, vasculitis, congenital coronary anomaly, and embolism [8]. The most common are vasospasm, dissection and fibromuscular dysplasia.

**Figure 6:**
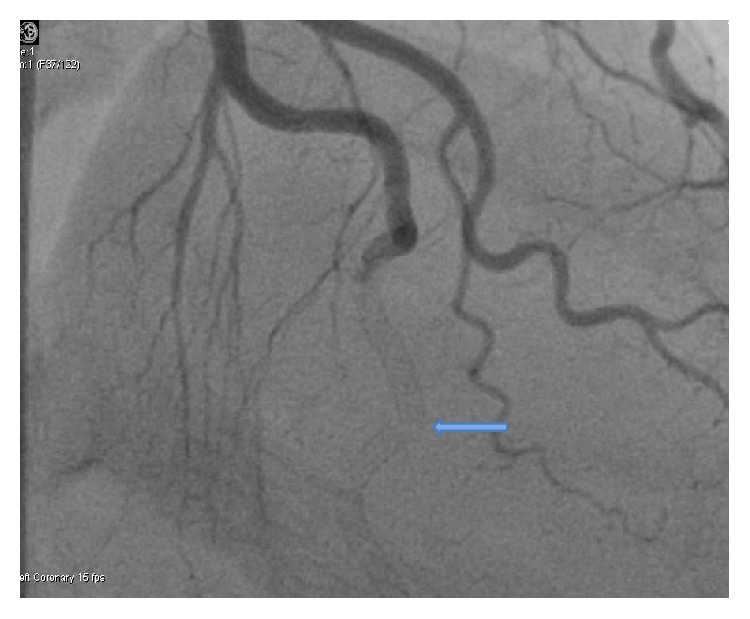
Possible stent fracture (arrow) in distal LAD during second catheterization.

During cardiac cath., her LAD did not dilate with intracoronary nitroglycerin administration, which makes coronary vasospasm less likely; no dissection was seen on the original study. The patient then had acute stent thrombosis within 15 hours of primary percutaneous coronary intervention, which may be related to the total amount of stent used (78 mm) or multiple stents in a small caliber system [9]. From the subsequent angiographic appearance and the inability to safely pass a guidewire and 1.5 mm balloon, stent fracture (Figure 6) resulting in acute stent thrombosis is most likely.

In this case she has hypertension as her only coronary risk factor. She presented on leuprolide, which is known to decrease estrogen to postmenopausal levels. Estrogen has a cardioprotective role, regulating lipid profile and improving endothelial function [10]. A few studies suggested that estrogens increase endothelium-nitric oxide synthase, which subsequently increases the production of nitric oxide as an endothelium-derived relaxing factor [11]. It was reported that, in premenopausal women, estrogen administration improved endothelium dependent coronary vasodilator function, manifested as increased coronary flow [12]. Leuprolide could have been the culprit for her myocardial infarction and her early STE was probably due to strut fracture.

## References

[1] Lloyd-Jones D., Adams R. J., Brown T. M. (2010). Heart disease and stroke statistics—2010 update: a report from the American Heart Association. *Circulation*.

[2] Goldberg R. J., McCormick D., Gurwitz J. H., Yarzebski J., Lessard D., Gore J. M. (1998). Age-related trends in short- and long-term survival after acute myocardial infarction: a 20-year population-based perspective (1975–1995). *The American Journal of Cardiology*.

[3] Barrett-Connor E., Bush T. L. (1991). Estrogen and coronary heart disease in women. *The Journal of the American Medical Association*.

[4] Davis C. E., Pajak A., Rywik S. (1994). Natural menopause and cardiovascular disease risk factors: the Poland and US collaborative study on cardiovascular disease epidemiology. *Annals of Epidemiology*.

[5] Levine G. N., D'Amico A. V., Berger P. (2010). Androgen-deprivation therapy in prostate cancer and cardiovascular risk: a science advisory from the American Heart Association, American Cancer Society, and American Urological Association: endorsed by the American Society for Radiation Oncology. *Circulation*.

[6] Sasaki T., Kurosawa T., Yamaguchi H. (2010). Myocardial infarction in a premenopausal woman with a decreased serum estrogen level due to leuprorelin acetate. *Journal of Cardiology Cases*.

[7] McCoy M. J. (1994). Angina and myocardial infarction with use of leuprolide acetate. *The American Journal of Obstetrics and Gynecology*.

[8] Saw J., Aymong E., Mancini G. B. J., Sedlak T., Starovoytov A., Ricci D. (2014). Nonatherosclerotic coronary artery disease in young women. *The Canadian Journal of Cardiology*.

[9] Aoki J., Lansky A. J., Mehran R. (2009). Early stent thrombosis in patients with acute coronary syndromes treated with drug-eluting and bare metal stents: the acute catheterization and urgent intervention triage strategy trial. *Circulation*.

[10] Bush T. L., Barrett-Connor E., Cowan L. D. (1987). Cardiovascular mortality and noncontraceptive use of estrogen in women: results from the lipid research clinics program follow-up study. *Circulation*.

[11] Simoncini T., Hafezi-Moghadam A., Brazil D. P., Ley K., Chin W. W., Llao J. K. (2000). Interaction of oestrogen receptor with the regulatory subunit of phosphatidylinositol-3-OH kinase. *Nature*.

[12] Reis S. E., Gloth S. T., Blumenthal R. S. (1994). Ethinyl estradiol acutely attenuates abnormal coronary vasomotor responses to acetylcholine in postmenopausal women. *Circulation*.

